# Is HER2 amplification predictable by digital immunohistochemistry?

**DOI:** 10.1186/1746-1596-8-S1-S14

**Published:** 2013-09-30

**Authors:** Tamás Micsik, Gábor Kiszler, Daniel Szabó, László Krecsák, Tibor Krenács, Béla Molnár

**Affiliations:** 1 Ist Department of Pathology and Experimental Cancer Research, Semmelweis University, Budapest, Hungary; 2 3DHistech Ltd., Budapest, Hungary; 3 H-1063 Budapest, Podmaniczky u. 63, Hungary

## Background

In the last decade anti-HER2 treatment became one of the best examples for targeted treatment. Since the aggressive behavior of HER2-positive breast cancers could have been successfully reduced by trastuzumab therapy, HER2 positive breast cancers recently show improving prognosis. According to a four-tiered classification of international clinical guidelines, cases with strong and complete staining (IHC 3+) with anti-HER2 antibodies are eligible for trastuzumab therapy. The cases with complete, but moderate anti-HER2 stainings (2+ or equivocal) should be further investigated with (F)ISH-technique to determine HER2-amplification [1]. Negative and IHC 3+ cases are easy to interpret semi-quantitatively on routine immunohistology, it is hard to conclude on the equivocal cases, sill, anti-HER2 therapy is indicated upon the predictive pathology report of HER2-expression and interobserver variability of IHC-interpretation still remains rather high [2]. Furthermore, the response rate of patients to the rather expensive tratuzumab therapy that might be accompanied by side effects is still only about 50% [3]. The rapidly developing digital pathology solutions have promised better ways of archiving, documenting and standardizing immunohistochemistry including image analysis of HER2 detection to improve the efficacy of targeted anti-HER2 therapy [4].

*MembraneQuant* application of *Pannoramic Viewer* platform (3DHISTECH, Budapest, Hungary) offers standardized way for semi-automated scoring of membrane-staining. Our aim was to validate *MembraneQuant* application against semi-quantitative routine scoring of HER2 IHC slides in order to improve prediction of HER2 gene amplification status.

## Materials and methods

### Patients

*We selected invasive breast cancers from year 2002-to 2005 from the* archive of the 1st Department of Pathology and Experimental Cancer Research of the Semmelweis University, Budapest, Hungary. 100 invasive ductal carcinomas were used in TMAs (created with *TMA Master*, 3DHISTECH Ltd, Budapest, Hungary) of 2mm cores of formalin-fixed paraffin embedded breast cancer specimens from females aged 26-86 years. The survey was performed with the permission of the *Ethical Committee*. TMA slides were used for HER2 IHC according to manufacturer’s protocol on a Bond-max^TM^ fully automated staining system (Leica Microsystems GmbH, Germany), using PATHWAY® HER-2/*neu* (clone 4B5, Ventana, USA), whereas their duplicates were used for HER2-FISH testing by the Rembrandt Her2/Neu - Cen 17 FISH kit (PP-C801K.5206, Biomedica kft. Budapest).

### Digitalization platform

Digitalization was performed using a *Pannoramic SCAN* digital slide scanner (3DHISTECH) using Zeiss plan-apochromat objective (magnification: 20X, Numerical aperture: 0.8) and Hitachi (HV-F22CL) 3CCD progressive scan color camera (resolution: 0.2325 µm/pixel). For fluorescent slides Zeiss (AxioCam MRm Rev. 3) monochrome microscopy camera (resolution: 0.322500 µm/pixel) was used which has high spectral sensitivity.

### Digital evaluation of slides

In our application a color deconvolution algorithm was applied to separate the signal of immunoreactive cell membranes in the brown channel (DAB signal) from the counterstain blue channel (hematoxilin signal). The color deconvolution algorithm generates two different grayscale images which are separately processed. The membrane detection algorithm runs on the brown, whereas the cell nuclei detection on the blue channel. The immune-negative epithelial cells have no membrane stain so these cells are to be detected on their cell nuclei on hematoxilin signal. The processing of the cell nuclei segmentation is similar to the *NuclearQuant* application which has been previously described [5].

Cell membrane immunostained slides can be described as connected pixel curve of local minima of intensity in DAB image. The intensity based linking algorithm was developed to segment the image into adjacent spots whose border potentially marks the middle of the membranes lines (skeleton). Some false curves can appear at local minima where is no actual immunreaction presented, therefore adjacent spots which have suboptimal features should be merged. Merging criteria are based on area of the spots and the length of neighboring border segments. Further false curves can be eliminated based on nuclei segmentation: adjacent spots are merged which has common border segment over a nuclei. After successful subtraction of membrane and nuclei segmented mask images spots are disclosed which could not represent membranes based on their size. Average intensity of DAB image is measured along the border of spot locations to be used for classification (scoring) [6].

**Figure 1:**
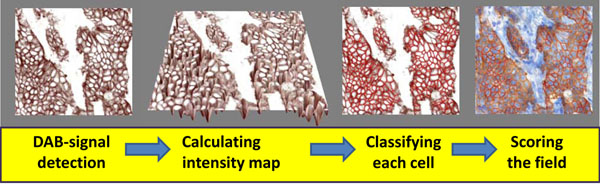
**Digital analysis of scanned slides** The DAB-imunostaining levels are detected on the brown-channel. MembraneQuant detects the nuclei on the blue channel to identify the cells of the ROI, than calculates an intensity topographic map to classify each cell to a distinct class, which is later on calculated to a field score (here the strong, complete membrane staining was of a 3+ score). On the result-slide the positivity-class of the cells are color-coded (blue: nucleus, yellow: 1+, orange: 2+, red: 3+).

*MembraneQuant* detects all cells and counts individually its specific staining in a region of interest (Figure 1). These proportional and intensity data later combined to a Field Score according to the guidelines, but all other data can be extracted from the digital analysis (e.g. H-Score, Label area, mask area, number of detected objects).

### Semiquantitative-scoring of the slides

Visual scoring of the digitized HER2 slides was performed blinded with regards of the original HER2 scores. A pathologist reviewed the digital TMA-cores using *Pannoramic Viewer* application (3DHISTECH) and provided a HER2 score and selected 1 to 4 annotations of tumorous tissue as regions of interest (ROI) on each core and scored them individually. Altogether 226 annotations were selected and analyzed using *MembraneQuant* and the scores of the ROI on one core were summarized into an overall core score, which was used for the data analysis.

FISH scoring and settings of *MembraneQuant* were equivalent to the international HER2- ASCO/CAP scoring guidelines [1]. Data analysis of the immunreaction was performed using the Statistica 9.1 (StatSoft Inc, Tulsa USA).

Agreement between the different scorings was calculated using Cohen’s kappa. The strength of agreement was interpreted as proposed by Landis & Koch [7]. In order to test the clinical relevancy of the agreement, quadratic weighted kappa was calculated as well, by assigning the following weights: 1 for total agreement; 0.89 for 0 vs. 1+ or 2+ vs. 3+ or 1+ vs. 2+; 0.56 for 0 vs. 2+; 0 for 0 vs. 3+. the weight 0 for the most relevant disagreement (i.e. 0 for 2+ vs. 3+). The strength of the agreement was additionally assessed using the Spearman rank-correlation coefficient.

## Results and discussion

Cohen’s kappa revealed an almost perfect agreement (κ = 0.857, 95%CI = 0.750-0.929) between the scores provided by the pathologists and those by *MembraneQuant*. While testing the agreement for clinical relevancy, this proved to be an almost perfect correlation, as showed by the high quadratic weighted kappa value (κ = 0.962, 95%CI = 0.939-0.986). Spearman rank-correlation also provided a highly significant correlation between the results (Spearman's rho = 0.933, df = 99, p < 0.0001, 95% CI for rho 0.903-0.955).

**Figure 2:**
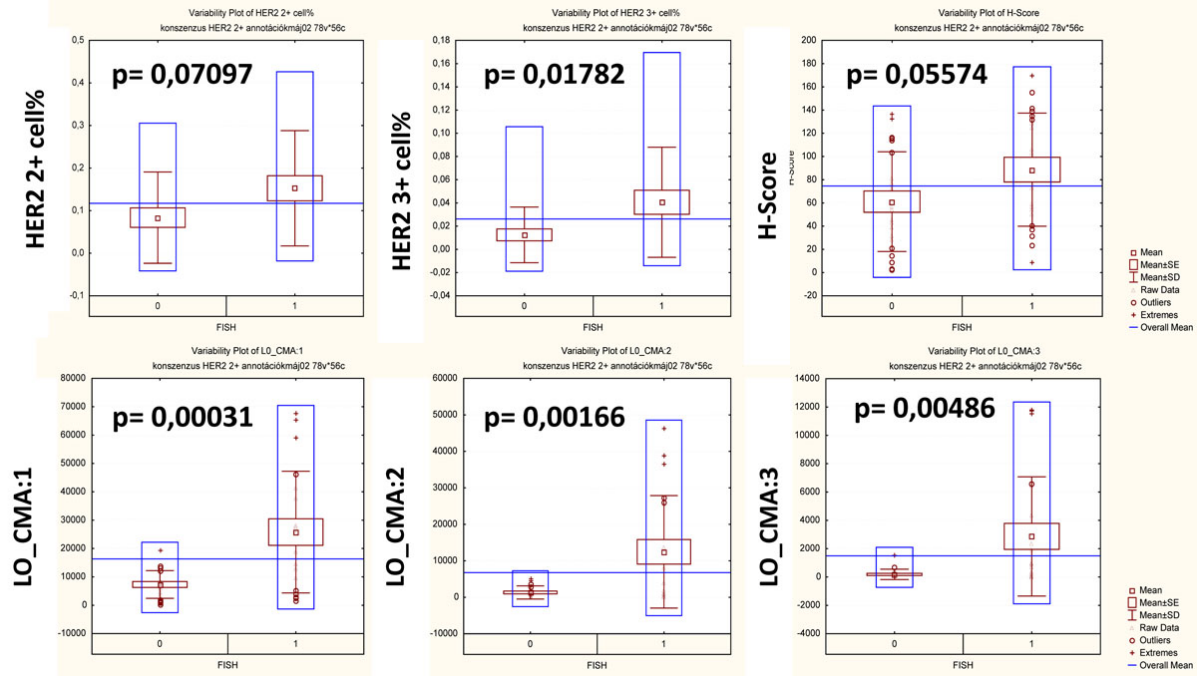
**Graphs of significant findings according to FISH-positivity.** Legend: According to data in Table 1. significant findings were visualized with variability plots and p-values are indicated in each graph. Most significant differences were found in the Label Area and within the HER2 3+ proportional score, while strong tendency was found in H-Score according to FISH-positivity. Only trend was found in HER2 2+ proportional score.

In the 15 equivocal cases 9 were found FISH-, while 6 were FISH+. During digital processing of IHC-slides MembraenQuant calculates different values for each cell, which data were later analyzed in all the FISH tested cases in order to predict HER2 amplification status. There was a trend towards lower HER2-negative cell number and higher 2+ cell number in FISH-ve cases, while FISH+ve cases had significantly higher 3+ cell number. By multiplying the frequency of positive cells with the class of IHC-positivity given by the software we calculated the H-Score for all ROIs and found a borderline significant difference between the FISH+ve and FISH-ve cases with elevated H-score in the FISH+ cases. Among the data counted by the *MembraneQuant* we also found other significant differences and one of the most promising was within the ’mask area of objects in different classes’ (LO-CMA) to differentiate between FISH+ve and FISH-ve cases (Table 1 and Figure 2).

**Table 1:** Differences in membrane-stainings according to FISH-positivity.

	FISH -	FISH +	FISH -	FISH +	FISH -	FISH +	FISH -	FISH +	FISH -	FISH +	FISH -	FISH +
	
p=	***0.07097***		0.01782		***0.05574***		0.00031		0.00166		0.00486	
	
	HER2 2+ % RATIO	HER2 2+ % RATIO	HER2 3+ % RATIO	HER2 3+ % RATIO	H-Score	H-Score	L0_CMA:1+	L0_CMA:1+	L0_CMA:2+	L0_CMA:2+	L0_CMA:3+	L0_CMA:3+
**Average**	**0.08386**	**0.152766**	**0.012607**	**0.04071**	**61.25344**	**88.80895**	**7315.882**	**25794.81**	**1317.471**	**12464.48**	**184.7127**	**2866.724**
SD	0.107272	0.135455	0.024028	0.047413	42.95535	48.74765	4898.122	21455.78	1796.785	15429.72	365.2118	4210.592
Min.	0	0	0	0	3.007519	9.550562	207.6841	1472.817	0	0	0	0
**Max.**	0.28777	0.408517	0.098592	0.162461	**136.6906**	**170.5047**	19422.9	67658.91	5221.888	46501.73	1549.739	11813.45

*MembraneQuant* calculates intensity scores for each cell within an annotation and additional proportional score. Significant difference is highlighted underlining; italics indicates strong tendency. FISH+ cases had significantly higher HER2-IHC3+ proportional score, while IHER2-IHC2+ proportional score and H-score had a tendency of higher occurrence in the FISH+ cases. In our set, the most significant differences (underlined) were found between FISH-positive and negative cases in the label-area of different IHC-intensity classes.

The anti-HER2 targeted therapy is indicated upon strong (3+) HER2-IHC staining which correlates well with the HER gene amplification measured by FISH [6]. FISH, however, requires special infrastucture accessed by only limited number of laboratories and several studies have concluded, that protein expression level might correlate better with the efficacy of trastuzumab therapy [3]. The four-tiered classification of HER2 immunohistochemsitry may not be a sufficient thumbrule and there is an increasing amount of information suggesting that a continous HER2-score might correlate better to the response to trastuzumab therapy [8]. In our approach, the different datasets generated during the processing of digital slides might help to better differentiate markers of HER2-amplification, which may not be evaluated consistently by the pathologist’s eyes during the semi-quantitative analysis. MembraneQuant application calculates the proportional score for each intensity class (0 to 3+) and also many other further derivatives on the annotated ROI areas related to our digital immunohistochemistry data, which may be used to validate HER2-IHC and its predictive value on HER2-amplification.

We found lower 0 proportional score, and higher 2+, 3+ proportional score and H-score in FISH-positive cases on a set of 15 equivocal cases further assesed with FISH. 3+ proportional score was significantly higher in FISH+ cases, and we also found more datasets (without known clinical meaning) derived by the algorythm, which showed highly significant differences in correlation with the FISH-findings. Althought, the negative and positive predictive values for reliable detection of FISH-amplification should be calculated on a higher sample number, our findings are promising since there are several other HER2 digital analysis platforms [8-10]. However, these platforms are based on more difficult and complex investigations and use more antibodies and fluorescent dyes. Our analysis relies on a standard quality IHC-reaction gained with a highly specific antibody (clone 4B5) and uses multiple factors to predict HER2-amplification.

## Conclusion

We validated *MembraneQuant* application of *Pannoramic Viewer* platform by finding an almost perfect correlation between digital and semi-quantitative evaluation of HER2-IHC slides (quadratic κ = 0.962, Spearman's rho = 0.933). Furthermore, we found several significant differences in the staining-patterns of the equivocal cases, which could help to discriminate between FISH-positive and negative cases by combining the 4-tiered classification with other digitally-derived sample data. We strongly believe, that digital image analysis methods - digital immunohistochemistry - can improve the efficacy of anti-HER2 therapy by standardizing the evaluation protocols and finding discriminative patterns within digital data sets to detect HER2-amplification.

## List of abbreviations

CCD: Charge Coupled Device; DAB: Diaminobenzidin; FISH: Fluorescens In Situ Hybridization; IHC: Immunohistochemistry; HER2: Human Epidermal growth factor Receptor 2; LO-CMA: Layer0 - Mask Area of objects in Class; TMA: Tissue Micro Array

## Competing interests

Tamás Micsik and Tibor Krenács are working as consultants for 3DHistech for 5 years.

László Krecsák was formerly an employee for 3DHistech.

## Authors’ contributions

TM: Pathologist, performed immunohistochemical evaluation, wrote the majority of article,

GK: Made digitization, dealed with *MembraneQuant*

DS: optimized digitization and digital evaluation, protocol

LK: performed the majority of statistical analysis

KT: worked as consultant during work

BM: worked as consultant during work, managed company affairs

## Ackowledgements

We are very thankful for Mrs. Renáta Kiss in helping digitization process and for Mrs Annamária Csizmadia for performing and helping to evaluate FISH-reactions.
